# An NGO-Implemented Community–Clinic Health Worker Approach to Providing Long-Term Care for Hypertension in a Remote Region of Southern India

**DOI:** 10.9745/GHSP-D-17-00192

**Published:** 2017-12-28

**Authors:** Sujatha Sankaran, Prema S Ravi, Yichen Ethel Wu, Sharan Shanabogue, Sangeetha Ashok, Kaylan Agnew, Margaret C Fang, Raman A Khanna, Madhavi Dandu, James D Harrison

**Affiliations:** a University of California at San Francisco, San Francisco, CA, USA.; b Tribal Health Initiative, Sittilingi, Tamil Nadu, India.; c St. John's National Academy of Health Sciences, Bangalore, Karnataka, India.

## Abstract

Poor blood pressure control results in tremendous morbidity and mortality in India where the leading cause of death among adults is from coronary heart disease. Despite having little formal education, community health workers (CHWs) are integral to successful public health interventions in India and other low- and middle-income countries that have a shortage of trained health professionals. Training CHWs to screen for and manage chronic hypertension, with support from trained clinicians, offers an excellent opportunity for effecting systemwide change in hypertension-related burden of disease. In this article, we describe the development of a program that trained CHWs between 2014 and 2015 in the tribal region of the Sittilingi Valley in southern India, to identify hypertensive patients in the community, refer them for diagnosis and initial management in a physician-staffed clinic, and provide them with sustained lifestyle interventions and medications over multiple visits. We found that after 2 years, the CHWs had screened 7,176 people over age 18 for hypertension, 1,184 (16.5%) of whom were screened as hypertensive. Of the 1,184 patients screened as hypertensive, 898 (75.8%) had achieved blood pressure control, defined as a systolic blood pressure less than 140 and a diastolic blood pressure less than 90 sustained over 3 consecutive visits. While all of the 24 trained CHWs reported confidence in checking blood pressure with a manual blood pressure cuff, 4 of the 24 CHWs reported occasional difficulty documenting blood pressure values because they were unable to write numbers properly. They compensated by asking other CHWs or members of their community to help with documentation. Our experience and findings suggest that a CHW blood pressure screening system linked to a central clinic can be a promising avenue for improving hypertension control rates in low- and middle-income countries.

## Abstract (precis)

Paid community health workers screened for hypertension in the community, referred cases to the clinic for diagnosis and initial treatment by a physician, and then monitored patients who had well-controlled blood pressure including dispensing maintenance medications prescribed by the physician. Blood pressure control was successful in the majority of such patients.

## INTRODUCTION

Over the past decade, there has been a dramatic increase in rates of coronary heart disease (CHD) in the developing world. This trend is expected to continue with a projected increase in rates of CHD in low- and middle-income countries by 6 million deaths over the next 20 years.^1^ Globally, hypertension is responsible for more cardiovascular disease and premature death than any other modifiable risk factor. Uncontrolled hypertension leads to ischemic heart disease, renal failure, and strokes. Of the 31.1% of adults in the world with hypertension in 2010, only 7.7% had their blood pressure controlled to a rate of less than 140 systolic over 90 diastolic.^2^ The global Prospective Urban Rural Epidemiology (PURE) study showed that rates of cardiovascular disease and death were higher in low-income countries compared with high-income countries, and that the lack of adequate risk-factor control in low-income countries was likely driving this difference.^3^

A shift in disease burden from communicable diseases to noncommunicable diseases, such as hypertension, diabetes, and myocardial infarction, has been documented in both urban and rural regions of India.^4^ In 2012, the prevalence of hypertension in rural India was between 15.4% and 21.9%.^5^ The South Asian cohort of the PURE study showed poor control of CHD risk factors, with more than 80.0% of patients with a history of CHD or stroke not receiving any protective drug therapy at the median of 4 years after diagnosis.^6^ Improved control of hypertension is vital in order to decrease overall rates of CHD mortality in low- and middle-income countries.^2^

Two of the major barriers to patients accessing health care in under-resourced regions are the lack of adequate numbers of providers in those regions and the challenge and cost of traveling from remote regions to available health care providers. Community health worker (CHW) systems, which train individuals from within villages to provide first-line care, have been successfully implemented for a variety of public health concerns, including childhood vaccinations, maternal and child health, and tuberculosis and HIV monitoring programs. CHW programs have been discussed as a way to improve the screening, diagnosis, and management of noncommunicable diseases in resource-poor settings.^7^ Accordingly, CHW programs in remote regions, where clinical services are difficult to access, hold potential for improving care of hypertension, especially because this condition requires frequent follow up.

Health workforce shortages and transportation costs are major barriers to patients accessing health care in under-resourced regions.

In September 2017, a cluster randomized controlled trial in Argentina showed that a CHW-led multicomponent intervention was superior to usual management of hypertension.^8^ An earlier study in India, published in 2010, showed that health aides in Tamil Nadu could accurately screen for hypertension and refer patients for hypertension management; however, patients were not followed longitudinally to determine whether adequate blood pressure control was achieved.^9^ A cluster randomized controlled trial conducted in 2009 in Karachi, Pakistan, showed that home health worker visits, coupled with education by physicians, were effective at decreasing rates of hypertension in the primarily urban and educated study population.^10^

The Sittilingi Valley is a remote, tribal region in the southern Indian state of Tamil Nadu with difficult-to-access dirt roads and limited options for public transportation. According to Indian government census data from 2011, the primary occupation of the Sittilingi Valley population is farming, the primary language is Tamil, and less than 10% of the population receives higher than a sixth-grade education.^11^ Accessing the central clinic is a major barrier for patients in this region, and providers at the central tribal clinic have had difficulty providing consistent hypertension management using only the central clinic as an access point for patients. Poor access and inadequate treatment are exacerbated by poor health literacy, patients' reluctance to seek care for asymptomatic conditions, and a culture that prioritizes health care for younger people. In this setting, a CHW model has the potential to provide a higher level of hypertension management to patients in the valley.

In this article, we describe the development, implementation, and initial achievements of a CHW hypertension program in this area of rural India.

## PROGRAM DESCRIPTION

### Setting

In August 2013, the University of California San Francisco (UCSF) and the Tribal Health Initiative (THI) established a partnership to implement and study the effects of adapting an existing CHW program to include hypertension management in the rural under-resourced tribal region served by THI.

A “tribal region” in India is a government designation for areas with populations that were historically marginalized and are now officially recognized by the government as neglected.

THI is an Indian nonprofit organization founded in 1994 by 2 Indian physicians committed to improving the health of the rural tribal population. It is fully nongovernmental, in the sense that it does not interface directly with any governmental system. When THI was first formed, there was no government clinic in the designated tribal region. Since then, a government clinic has been established 11 km outside the tribal area. However, very few patients seen at the THI clinics and none of the patients enrolled in the hypertension program also seek care at the government clinic. The organization now serves the 12,000 people who live in the 21 villages located in the tribal region of the Sittilingi Valley through their 24-bed tribal hospital and outpatient clinic. Approximately 8 physicians work at the tribal hospital and outpatient clinic.

THI also has a CHW program that trains selected women to provide basic medical care in the villages. A CHW is a married woman from each village—aged 30 to 40 years with at least a tenth-grade education—who was chosen by her fellow villagers. Each CHW receives 2 days of training every 2 weeks for 18 months on the basics of sanitation, hygiene, childbirth, nutrition, and methods of communicating this knowledge to the community through stories and songs. A total of 24 CHWs are currently employed by THI and are paid an hourly rate using local standards for competitive wage compensation. Every week, CHWs see patients for 6 hours in their village, providing immunizations for children, prenatal care to pregnant women, and advice, treatment, and follow-up care for adult patients with other medical concerns. Patients with more complex complaints are referred by the CHWs to a THI tribal clinic.

### Stepwise Community Health Worker Hypertension Program

**FIGURE 1.**
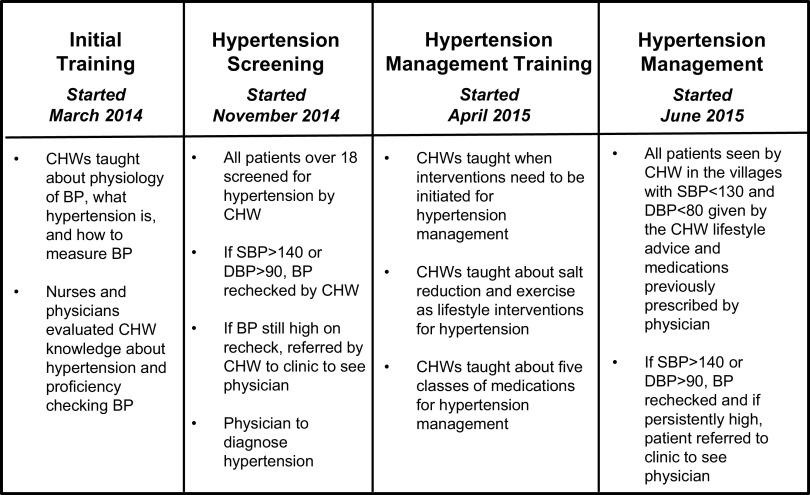
Stepwise Implementation of Community Health Worker Hypertension Program, Sittilingi Valley, Tamil Nadu, India Abbreviations: BP, blood pressure; CHW, community health worker; DBP, diastolic blood pressure; SBP, systolic blood pressure.

In January 2014, THI staff and UCSF faculty developed a curriculum for CHWs that teaches blood pressure measurement, diagnosis, management, and prevention based on the 2014 American Society of Hypertension and International Society of Hypertension (ASH/ISH) guidelines for hypertension management.^12^ The goal of the curriculum was to teach CHWs how to incorporate blood pressure measurement, documentation, and referral of hypertensive patients to the central clinic into their weekly responsibilities seeing patients in their villages each week. An additional 5 to 6 hours of work each week was allocated for this purpose. The training of the CHWs took place in a stepwise manner (Figure 1).

The goal of the curriculum was to teach CHWs how to incorporate blood pressure measurement, documentation, and referral of hypertensive patients to the central clinic.

#### Phase 1. Initial Training

Over a 7-month period, beginning in March 2014, the 24 CHWs at THI received monthly 3-hour hypertension training sessions led by the head nurse. The CHWs were taught about the physiology of blood pressure, what happens during systole and diastole parts of the cardiac cycle, and why measuring blood pressure is important. The CHWs also received 1 to 2 hours of practical training at the end of each session on the use of the Ashok Nisco-09 electronic arm blood pressure cuffs (Ashok Enterprises, Delhi, India), which record systolic and diastolic blood pressure and heart rate. They were also taught to record blood pressure and heart rate for each patient in a medical record. After the training was completed, each CHW's level of understanding of the training materials and equipment was evaluated by nurses and physicians observing a CHW's blood pressure measurement technique, checking their ability to record blood pressure numbers accurately, and assessing general understanding of what blood pressure is and why hypertension control is important. Two of the CHWs were found to have knowledge deficits and were required to undergo additional training to reinforce key points. Following completion of the training, the CHWs met monthly with the head nurse for continued mentoring and feedback about the CHW hypertension program.

#### Phase 2. Hypertension Screening in the Community

In November 2014, the CHWs began screening all patients over the age of 18 for hypertension during their weekly field clinics in the villages. The screening sites were located at a central setting in the village, usually a school or temple, where villagers would gather and meet with the CHW. The screening process was simple: a person would rest in a chair for 5 minutes with both feet on the ground, after which their blood pressure was measured. Anyone with either a systolic blood pressure greater than 140 or a diastolic blood pressure greater than 90 would receive a second blood pressure check. If the second check also was high, the patient was classified as having screened positive and was sent to the clinic, where he or she was seen by a physician. The physician would recheck the patient's blood pressure and, if it was elevated, the patient would receive a hypertension diagnosis and be enrolled in the hypertension program. From that point, the physician would manage the patient's hypertension in accordance to standard practices, providing lifestyle advice and medications, if warranted.

**FIGURE 2.**
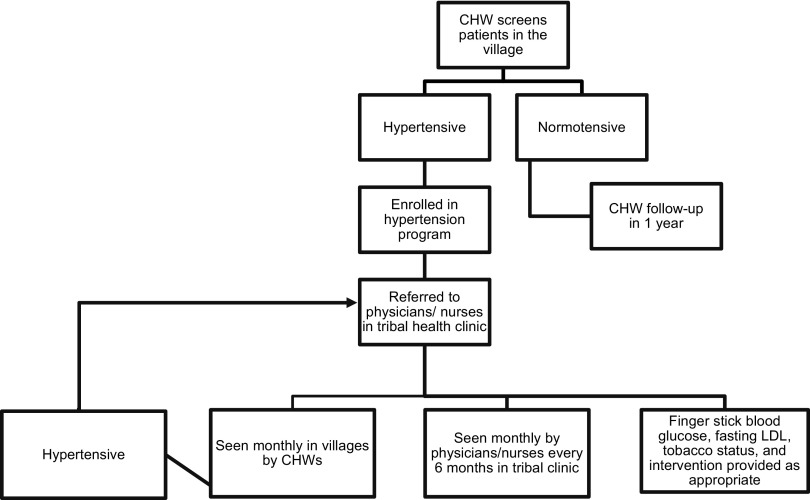
Community Health Worker Hypertension Program Screening Algorithm, Sittilingi Valley, Tamil Nadu, India Abbreviations: CHW, community health worker; LDL, low-density lipoprotein cholesterol.

During the first month following the screening event, a visiting nurse would monitor hypertensive patients every week. In the subsequent months, CHWs who had completed their first month of training screened patients for hypertension, recorded blood pressure values in a log, and referred patients for follow up either with the clinic (if screening suggested hypertension) or the CHW (if screening suggested normal blood pressure) (Figure 2). A visiting nurse would monitor patients' blood pressure measurements every 4 to 6 weeks and provide the CHWs with real-time feedback and additional teaching/mentoring.

A visiting nurse would monitor patients' blood pressure measurements every 4 to 6 weeks and provide the CHWs with real-time feedback.

A simple algorithm for hypertension management was formulated based on the 2014 ASH/ISH guidelines.^12^ In addition to measuring blood pressure, CHWs asked patients what antihypertensive medications they were taking, and documented this information in the record. During this phase of screening for hypertension, the CHWs continued to return to the clinic monthly for 2 hours of teaching, during which the head nurse reviewed hypertension management with the CHWs and answered questions that came up during their clinical work.

#### Phase 3. Training in the Management of Hypertension

In April 2015, the CHWs began to receive teaching during their monthly 2-hour sessions with the head nurse and clinic physician about hypertension management. Specifically, the head nurse and the clinic physician taught the CHWs about goal blood pressure ranges, the 5 classes of blood pressure medication available for patients (hydrochlorothiazide, amlodipine, lisinopril, metoprolol, and valsartan), potential side effects of the medication, and lifestyle interventions for blood pressure control.

The teaching augmented and reinforced the algorithm that CHWs had been trained to follow as to what blood pressure ranges and complaints necessitated immediate referral of patients to the clinic. The CHWs were also taught to refill medications with the same dosages for patients already on medication who were found to be normotensive during the field visits. They were also taught to refer all enrolled patients on medication to the clinic every 6 months for laboratory monitoring of kidney function and electrolytes and refer all enrolled patients not on medication to the clinic every year for routine laboratory monitoring.

While the CHWs received this more in-depth teaching monthly until October 2014, they continued to only screen patients for hypertension and send uncontrolled hypertensive patients to the clinic. The CHWs did not start providing any hypertension management interventions during the orientation period.

#### Phase 4. Management of Chronic Hypertensive Patients by Community Health Workers

**FIGURE 3.**
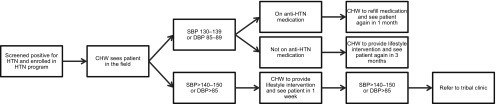
Community Health Worker Hypertension Program Clinical Decision Tree Algorithm, Sittilingi Valley, Tamil Nadu, India Abbreviations: CHW, community health worker; DBP, diastolic blood pressure; HTN, hypertension; SBP, systolic blood pressure.

In June 2015, the CHWs began giving lifestyle advice and dispensing medications for chronic conditions to patients with well-controlled blood pressure, based on a decision tree (Figure 3). The lifestyle advice that the CHWs provided to patients included restricting the amount of salt in their diets and performing moderate physical activity daily for 30 minutes. Patients on medication with well-controlled blood pressure, defined by a systolic blood pressure less than 130 and a diastolic blood pressure less than 80, were given maintenance medication by the CHWs during their monthly blood pressure check. For these patients, CHWs were able to continue to dispense previously prescribed medication to the patient without first consulting the physician. The CHWs never dispensed any new medications or new medication levels on their own. The CHWs referred patients with a systolic blood pressure greater than 130 or a diastolic blood pressure greater than 80 to the clinic in accordance with their prior teaching.

### Supervision of the Community Health Workers

Supervision of the CHWs began well before the initiation of the hypertension program. Visiting nurses made trips to each village every 2 months, to observe and supervise CHWs providing clinical care and to address illness and treatment management concerns of the villagers that could be taken care of by the CHWs. Additionally, the nurses provided ongoing trainings and educational meetings with CHWs based on deficiencies observed in the field. Once every 6 months, a physician would accompany the visiting nurses to the villages in order to provide additional CHW observation, on-the-ground training, and quality assurance. After the hypertension program was initiated, the visiting nurse and physician supervision was expanded to assess blood pressure measurement, management, and referral by CHWs.

### Consent, Screening, and Clinical Documentation

Before screening village members, each CHW asked all patients over the age of 18 for consent using a verbal consent script that explained in Tamil the purpose of the intervention and the potential harms. All patients who gave verbal consent were screened for hypertension. Documentation of patient care began with assessments by CHWs. Once measured, a patient's blood pressure value was written on a white medical record card by the CHW and given to the patient. All screened patients, regardless of blood pressure level, were given a white card. If the patient screened positive for hypertension they were given a referral to see a physician at the clinic and were told to bring their medical record card with them to all visits because a clinician would not be able to see them unless they had their card with them. The referral included both a verbal notification that the patient needed to be seen at the clinic and a written note stating this fact. When the patient arrived at the clinic, the clinic administrative assistant would transcribe the date and blood pressure value from the patient's white medical record card on to a blue medical record card. The blue card is the patient's hypertension record for the clinic, which stays in the clinic. When patients are seen in the clinic, blood pressure values from the clinic visit are entered onto both the white and blue medical record cards. Weekly, the clinic staff enters blood pressure data from the blue cards into the computer system; the data are then uploaded onto a secure server for access by authorized local and UCSF program personnel using a password-protected front end.

### Care in the Clinic

Patients who screened positive for hypertension were sent to the clinic, where they received care from 1 of 4 physicians who rotated through the clinic. The physician rechecked blood pressure and diagnosed hypertension in patients with systolic blood pressure over 140 or diastolic blood pressure over 90 in accordance with the ASH/ISH 2014 guidelines. Patients diagnosed with hypertension were enrolled in the hypertension program and seen by the physician weekly until blood pressure control was achieved. All patients in the program were also screened for diabetes and received baseline laboratory chemistry panels and blood urea nitrogen and creatinine levels. Patients who screened positive for diabetes or chronic kidney disease were managed in the clinic for these conditions. All patients enrolled in the hypertension program were referred to the clinic every 6 months to 1 year in order to check routine lab work. Each year, physicians attend an hour-long refresher lecture from a visiting UCSF faculty member about hypertension diagnosis and management guidelines.

**Figure:**
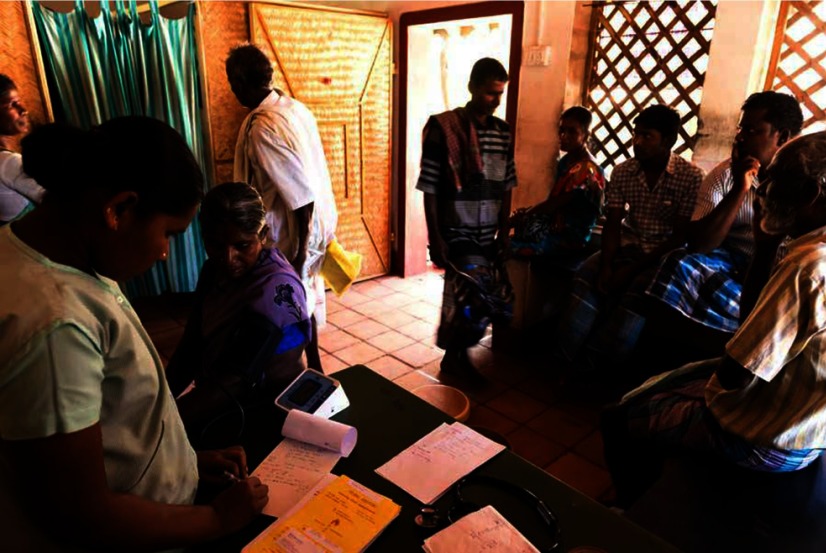
Clinic staff members see patients enrolled in the hypertension program. © 2017 Tribal Health Initiative.

After blood pressure control was achieved, a patient would be seen by their CHWs for monthly blood pressure checks. If the blood pressure remained within goal range during the CHW visits, the CHW would continue to provide lifestyle advice and medications as outlined above. If the blood pressure was elevated during a visit, the patient would be referred back to the clinic. Communication between CHWs and physicians occurred monthly, when the CHWs would visit the clinic for ongoing education, feedback, and communication of any patient-specific concerns with the physicians.

Communication between CHWs and physicians occurred monthly, when the CHWs would visit the clinic for ongoing education, feedback, and communication of any patient-specific concerns.

### Medication Recording

Beginning in June 2015, THI trained CHWs to document the medications using a color code—each medication having its own color sticker. When a physician, nurse, or CHW dispensed medication, the same color sticker would be placed on the patient's white medical record card. The CHW subsequently would check to ensure that the medication she is dispensing has the same color code as the medication the patient is already on. This acts as a safety measure to ensure that the correct medication is given to the patient, and is particularly important because 7 of the 24 CHWs are semiliterate and not able to read or document numbers accurately some of the time. Because the semiliterate CHWs initially had trouble documenting numbers correctly, the head nurse and visiting nurses spend an additional hour at the end of each monthly teaching session with these CHWs to teach them how to document blood pressure values correctly. When visiting nurses observed the CHWs in the field, their subjective observations suggested no difference in the care rendered by the semiliterate and literate CHWs.

## METHODS

**FIGURE 4.**
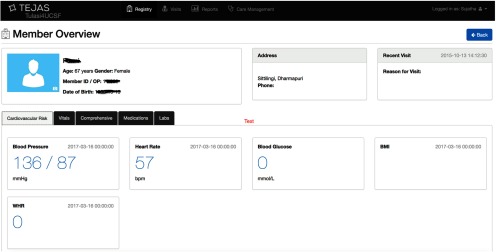
Screenshot of the Front-End Interface Used by Clinic Personnel to Enter Patient Demographic and Clinical Data for the Hypertension Program

Patient demographic and clinical data, including blood pressure data, from the patient blue cards were entered by clinic personnel through a front-end interface (Figure 4) and stored on a secure HIPAA (Health Insurance Portability and Accountability Act)-compliant virtual server developed by an Indian nonprofit organization, Tulasi. The server only stored patient data from hypertensive patients. Information about patients who did not screen positive for hypertension was kept by the CHW and later given to study personnel. Patient data recorded by the CHW at each follow-up visit were recorded on the white card, and entered into the server when the patient was seen at the clinic at least once every 6 months. The data were audited monthly to determine population hypertension control rates, demographic distribution, medication use, and time to control.

Regular informal feedback sessions were held separately with the head nurse, all 24 CHWs at the central clinic, all THI physicians, and a subset of patients who volunteered to talk to the program team. During these feedback sessions, participants discussed their perceptions of the hypertension program, program strengths and weakness, areas for improvement, ways in which the program benefitted or harmed the community, and thoughts about the future trajectory of the program. No formal qualitative data analysis or audio recording were completed during the informal feedback sessions, but we provide a summary of key comments expressed during these sessions. Informal oral patient questionnaires were conducted with 235 randomly selected patients enrolled in the hypertension program 1 year after the program began

### Ethical Approval

The University of California San Francisco Institutional Review Board and Indian Ethics Committee approved this program.

## PROGRAM ACHIEVEMENTS

The program had 3 main objectives: (1) a majority of community members over the age of 18 should be screened for hypertension by CHWs, and hypertensive patients were to be enrolled in the program; (2) continuing care should primarily occur in the villages by CHWs, while patients with uncontrolled hypertension should be referred to the clinic; and (3) hypertension control rates should be non-inferior to clinic-based hypertension control rates.

### Screening

By 2016, at the 2-year assessment point, the program had met the first objective, with a majority of people over the age of 18 screened for hypertension and appropriate patients enrolled in the program. A total of 7,176 people over the age of 18 had been screened: 3,445 (48.0%) were women and 3,731 (52.0%) were men (Table). Of those screened, 3,936 were between 18 and 39 years old, 314 (8.0%) of whom were hypertensive; 2,124 were between 40 and 59 years old, 424 (20.0%) of whom were hypertensive; and 1,116 were 60 years old or above, 446 (40.0%) of whom were hypertensive. In total, 1,184 (16.5%) of the 7,176 screened were hypertensive.

**TABLE.** Hypertension Rates in Patients Over Age 18 Screened by Community Health Workers, Sittilingi Valley, Tamil Nadu, India, 2014–2016

	Age Group (in Years)			Total
	18–39	40–59	≥60	
Hypertensive^a^	314 (8.0)	424 (20.0)	446 (40.0)	1184 (16.5)
Normotensive^b^	3622 (92.0)	1700 (80.0)	670 (60.0)	5992 (83.5)
Total	3936 (100.0)	2124 (100.0)	1116 (100.0)	7176 (100.0)

All data are shown as No. (%).

a Systolic blood pressure >140 or diastolic blood pressure >90 on 2 checks.

b Systolic blood pressure <140 and diastolic blood pressure <90

At the 2-year assessment point, CHWs had screened more than 7,000 people for hypertension, and about 17% were hypertensive at baseline.

### Community-Based Care

The program staff were able to successfully meet the second objective of training the CHWs to perform hypertension management in the field, with chronic care being provided in the community by CHWs, and the clinic being use primarily for care of uncontrolled hypertension. The main outcome assessed was whether CHWs were able to effectively measure and document blood pressure, refer patients with elevated numbers to the physician at the clinic, and provide continuing interventions that were previously given by a physician to patients with well-controlled blood pressure. We were not assessing how well the CHWs were controlling blood pressure, but rather that they were able to successfully implement the system. While all of the 24 CHWs successfully completed training, the 7 semiliterate CHWs needed additional training with the nurses in order to achieve competence. The CHWs were trained to check blood pressure, document blood pressure values, provide lifestyle counseling and medication, and refer patients to the clinic as needed.

The competence of the CHWs to check blood pressure, document blood pressure values, provide lifestyle counseling and medications, and refer appropriate patients to the clinic, as described above, was repeatedly assessed with visiting nurse observations that initially took place weekly and later monthly, visiting physician observations twice annually, and monthly didactic sessions with a question-and-answer component at the central clinic. Initially, 4 of the 24 CHWs encountered occasional difficulty recording blood pressure values, but by the end of the training period, these difficulties improved and the visiting nurses assessed all CHWs as competent. Confidence of the CHWs was assessed by monthly feedback sessions with the nurses and physicians at the central clinic.

The CHWs reported varying degrees of confidence in their ability to do their job. When the program began, many of the CHWs felt intimidated by the new skills they were asked to master, but after 1 year of implementation, all the CHWs said they felt confident checking blood pressure with a manual blood pressure cuff. Four of the 24 CHWs reported occasional difficulty documenting values because they were unable to write numbers properly. These CHWs reported that they asked other CHWs or members of their community to help with documentation. None of the CHWs expressed uncertainty or difficulty with dispensing medication. The CHWs expressed satisfaction with their work during informal feedback sessions and expressed interest in continuing the work in the future.

During one feedback session, a CHW stated, “people in my village respect me now because they know I have knowledge.” Another CHW stated, “now people come to me for help. They don't feel so nervous about being so far from the hospital. I'm here to calm them down. This makes me feel good.” The main challenge CHWs cited was their inability to convince patients to take directed advice. As one CHW stated, “[patients] don't believe me. I tell them not to eat salt but they do anyway.” As the program progressed, THI administration was able to incorporate CHW roles and responsibilities into the work they were already performing in the community.

During one feedback session, a CHW stated, “people in my village respect me now because they know I have knowledge.”

While CHWs were successfully trained to manage hypertension in the field, feedback from CHWs and patients revealed that the reasons a patient chose to use the clinic instead of community-based care were highly subjective and patient-specific. Informal oral patient questionnaires were conducted with 235 randomly selected patients enrolled in the hypertension program 1 year after the program began. The respondents were asked 2 open-ended questions: (1) “Are you satisfied with the care you are receiving through the hypertension program?” and (2) Do you visit the tribal clinic for hypertension only when directed by your CHW or at other times as well?” Of these respondents, 96 patients (40.9%) expressed satisfaction with the CHWs and stated that they only chose to visit the clinic when directed by the CHW or if they were experiencing an urgent concern. However, 28 patients (11.9%) expressed concern that the care they were receiving from the CHW was inferior to physician and nursing care, and reported visiting the clinic for hypertension management even when their CHW told them it was not necessary. When checked against clinic program data, 165 of the respondents (70.2%) had poorly controlled hypertension, while 70 (29.8%) had well-controlled hypertension. Of the 70 patients, 64 were recorded as having had well-controlled hypertension on their previous CHW encounter in the community, implying that their use of the clinic was due to patient preference as opposed to CHW referral.

### Hypertension Control Rates

For the third objective of achieving hypertension control rates that were non-inferior to clinic-based hypertension programs, we found that after 2 years of the program, 898 of 1,184 (75.8%) patients diagnosed as hypertensive achieved blood pressure control, which is defined as a systolic blood pressure less than 140 and a diastolic blood pressure less than 90 sustained over 3 consecutive visits either at the clinic or in the community. An average of 68 days or 2.9 visits were needed to first achieve blood pressure control in these patients with hypertension.

## DISCUSSION

We describe a program that used an existing CHW system linked to a central clinic as a mechanism to provide management of hypertension in a remote under-resourced community. We found that after 2 years, this program screened, enrolled, diagnosed, and managed the population of patients with hypertension and met expected objectives. This work demonstrates a feasible model for providing diagnosis and management of chronic conditions that consists of: (1) deliberate stepwise training and supervision of CHWs, (2) empowerment of CHWs with new skills of value to the community, (3) design of a CHW program that includes maintenance and follow up of a chronic condition, and (4) close integration of the CHW program with a clinic and referral system. The training program that was designed for our cohort of CHWs was delivered in separate components—to first screen patients for hypertension, then refer appropriate patients to the central clinic, and lastly manage chronic hypertensive patients in the field—in order to build CHW competence and confidence. From the qualitative data from our CHW training program, we found the CHWs were confident about their ability to do the separate components and found value in their work.

This work also demonstrates that using a CHW system linked to a central clinic can be an effective mechanism to shift chronic care management from physicians and nurses to CHWs, despite the subset of patients who seemed to prefer clinic-based care. We were able to show hypertension control rates of 75.8% in this community after 2 years were higher than the approximately 50% control rates reported in the general U.S. population,^13^ and considerably higher than those found in samples of the general Indian population, which are approximately 10% and 20% for rural and urban populations, respectively.^14^

Using a CHW system linked to a central clinic can be an effective mechanism to shift chronic care management from physicians and nurses to CHWs.

While our program was successful, 24.2% of patients identified as hypertensive did not achieve control within 2 years—this may be attributed to lack of adherence to medications, refractory hypertension, or lifestyle factors that were not addressed. Further study is needed to better understand factors related to poor control. We acknowledge that many factors contribute to achievement of blood pressure control, including dietary modification, exercise, adherence to medications, and trust in the health care system. Economic analyses are also needed to determine the cost differential between the CHW program and a physician-staffed clinic program, as the cost of long-term care is a known barrier to CHD risk-factor control in low- and middle-income countries.^15^ A CHW intervention may be effective at establishing an intact system, but these other factors must also be addressed in order to achieve optimal control. Future work examining the extent to which this CHW program increases access to hypertension care in remote, rural villages will further elucidate the benefits of this program and will help inform how this program can be used by ministries of health to improve hypertension care in remote areas.

In conclusion, our program suggests that a CHW system linked to a central clinic is a promising avenue for achieving improvements in hypertension control rates in low- and middle-income countries.

## Notes

**Peer Reviewed**

**Funding:** University of California San Francisco Division of Hospital Medicine and the International Cardiovascular Health Alliance.
